# Thermoelectrically-Cooled InAs/GaSb Type-II Superlattice Detectors as an Alternative to HgCdTe in a Real-Time Mid-Infrared Backscattering Spectroscopy System

**DOI:** 10.3390/mi11121124

**Published:** 2020-12-18

**Authors:** Raphael Müller, Marko Haertelt, Jasmin Niemasz, Klaus Schwarz, Volker Daumer, Yuri V. Flores, Ralf Ostendorf, Robert Rehm

**Affiliations:** Fraunhofer Institute for Applied Solid State Physics IAF, Tullastraße 72, 79108 Freiburg, Germany; marko.haertelt@iaf.fraunhofer.de (M.H.); jasmin.niemasz@iaf.fraunhofer.de (J.N.); klaus.schwarz@iaf.fraunhofer.de (K.S.); volker.daumer@iaf.fraunhofer.de (V.D.); yuri.flores@iaf.fraunhofer.de (Y.V.F.); ralf.ostendorf@iaf.frauhofer.de (R.O.); robert.rehm@iaf.fraunhofer.de (R.R.)

**Keywords:** InAs/GaSb, T2SL, IR, photodetector, TE-cooled, spectroscopy, RoHS, MCT

## Abstract

We report on the development of thermoelectrically cooled (TE-cooled) InAs/GaSb type-II superlattice (T2SL) single element infrared (IR) photodetectors and exemplify their applicability for real-time IR spectroscopy in the mid-infrared in a possible application. As the European Union’s Restriction of Hazardous Substances (RoHS) threatens the usage of the state-of-the-art detector material mercury cadmium telluride (MCT), RoHS-compatible alternatives to MCT have to be established for IR detection. We use bandgap engineered InAs/GaSb T2SLs to tailor the temperature-dependent bandgap energy for detection throughout the required spectral range. Molecular beam epitaxy of superlattice samples is performed on GaAs substrates with a metamorphic GaAsSb buffer layer. Photolithographic processing yields laterally-operated T2SL photodetectors. Integrated in a TE-cooled IR detector module, such T2SL photodetectors can be an alternative to MCT photodetectors for spectroscopy applications. Here, we exemplify this by exchanging a commercially available MCT-based IR detector module with our T2SL-based IR detector module in a real-time mid-infrared backscattering spectroscopy system for substance identification. The key detector requirements imposed by the spectroscopy system are a MHz-bandwidth, a broad spectral response, and a high signal-to-noise ratio, all of which are covered by the reported T2SL-based IR detector module. Hence, in this paper, we demonstrate the versatility of TE-cooled InAs/GaSb T2SL photodetectors and their applicability in an IR spectroscopy system.

## 1. Introduction

In numerous applications in science and industry, detection of infrared (IR) radiation is indispensable. A wide area of application is IR spectroscopy in the mid-infrared (MIR, 3–12 µm). Since several substances in gaseous, liquid, and solid state of aggregation have their characteristic transitions here, this region, which is sometimes referred to as the “fingerprint region”, is therefore clearly relevant for industrial or medical spectroscopy applications and when chemical identification or verification is required [1,2]. For industrial applications, common requirements of the IR detector arise. These can be summarized as: fast response, broadband spectral coverage, linearity, and high signal-to-noise ratio. These requirements can be met by specially designed IR photodetectors.

In an IR photodetector, a signal is generated after photon absorption across the fundamental bandgap of the underlying semiconductor material. The bandgap energy *E*_g_ of this material defines the cutoff wavelength of the detector, implying that radiation of longer wavelength cannot be detected. As the performance of IR photodetectors decreases for longer cutoff wavelength, choosing the detector cutoff wavelength based on the requirements of the application is essential. In general, cooling the detector material improves the performance of IR photodetectors. Utmost performance is achievable with expensive cooling with cryogenic liquids or Stirling coolers. However, for most applications, low-cost, small, lightweight IR detector modules are required. In these modules, the detector element is thermoelectrically cooled (TE-cooled) with multistage Peltier elements to a so-called high operating temperature (HOT) in the range between 180 K and 300 K.

So far, the commercialized state-of-the-art material of choice for HOT IR photodetectors is mercury cadmium telluride (HgCdTe or MCT). This is due to MCT featuring both a bandgap energy that is widely tunable in the IR, as well as a top-notch electrooptical performance. By adjusting the cadmium content, MCT allows for the fabrication of IR photodetectors with a cutoff wavelength in and beyond the fingerprint region. Numerous studies dedicated to the development and optimization of HOT MCT IR detectors have been conducted [3,4]. However, the Restriction of Hazardous Substances (RoHS) of the European Union regulates the allowed concentration of mercury and cadmium in electronic devices [5]. It is only due to temporary exemptions that this regulation does not prohibit the use of MCT detectors. Hence, for future applications, alternative, RoHS-compatible detector materials need to be established.

Since III-V semiconductors do not contain RoHS-restricted substances, RoHS-compatible photodetectors can be fabricated from them. For detection in the MIR, bulk III-V semiconductors are only partly suitable. InSb, the binary III-V material with the lowest bandgap energy, can only be utilized for detection up to around 5 µm when cryogenically cooled or up to around 7 micron for uncooled operation, which is insufficient for many applications. The ternary alloy InAs_1-x_Sb_x_ allows for bandgap tuning by modification of the composition. This enables bandgap energies that are smaller than the one of InSb. The limits of the bandgap tuning range for InAs_1-x_Sb_x_, i.e., the temperature and the composition dependence of the bandgap, were recently re-investigated [6]. As no substrate material exists that allows for lattice-matched growth of InAs_1-x_Sb_x_, handling the layer strain is inevitable.

**Figure 1:**
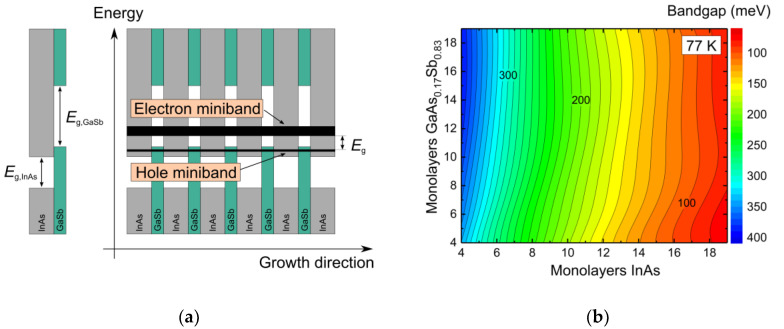
(**a**) Schematic of the type-IIb band alignment between InAs and GaSb and an InAs/GaSb type-II superlattice with lowest electron miniband and highest hole miniband. (**b**) Bandgap energy in dependence of the InAs/GaSb superlattice composition, calculated by the superlattice empirical pseudopotential method [10].

We investigate InAs/GaSb type-II superlattices (T2SLs) that are RoHS-compatible, feature a widely tunable bandgap energy and can be grown lattice-matched to GaSb [7,8,9]. InAs/GaSb T2SLs consist of alternating layers of InAs and GaSb that are usually grown by molecular beam epitaxy (MBE). Each individual layer is just a few atomic monolayers wide and acts as a quantum well for charge carriers. By quantum mechanical coupling of neighboring quantum well states, electron, and hole minibands are created, respectively. The fundamental bandgap of this artificial bandgap material opens between the lowest electron miniband and the highest hole miniband (see Figure 1a). It can be tuned by altering the width of the InAs and GaSb sublayers. Due to the peculiar type-IIb band alignment between InAs and GaSb, the superlattice bandgap energy can be engineered flexibly for a spectral range roughly corresponding to 3–20 µm, which is equivalent to photon energies from about 60 to 400 meV.

To illustrate the bandgap tuning in InAs/GaSb T2SLs, in Figure 1b a calculation of the bandgap energy in dependence of the superlattice composition based on the superlattice empirical pseudopotential method is shown [10]. Apparently, the InAs sublayer width has the main impact on the bandgap energy. Commonly, the superlattice composition is given in dependence of the sublayer width of InAs and GaSb, which is calculated based on calibrated growth rates and the MBE shutter sequence during T2SL growth. However, in Figure 1b, an As content of 17% is indicated for the GaSb sublayer, which was determined by X-ray diffraction. During the growth of a GaSb sublayer, the chamber atmosphere still contains As due to previously grown InAs sublayers. Since As is the group V component that is preferably incorporated into the layer, this leads to a non-negligible As content in the nominal GaSb sublayer. Details on the MBE growth procedure and the method for the determination of the As content in the GaSb sublayers are given in [10].

After the proposal to use InAs/GaSb T2SLs for IR detection [11], fundamental research on this material system [12,13] and development of single element detectors [14,15,16] and detector arrays [17] has intensified in the last decades. Important developmental steps in the field are reviewed in [18].

Activities in research and development of InAs/GaSb T2SLs have mainly focused on high-performance applications at low operating temperatures that require cryogenic cooling. As a result, for low operating temperatures, InAs/GaSb T2SLs emerge as a viable alternative to MCT for IR detectors and IR cameras. For the HOT range, IR detection with InAs/GaSb T2SLs in the longwave infrared was demonstrated [19,20,21,22], but dedicated device development and commercialization were never conducted. Now, mainly due to the RoHS, there is renewed interest in InAs/GaSb T2SL IR photodetectors for HOT applications.

Within the last few years, we have worked on the development of InAs/GaSb T2SL single element detectors for the HOT range and demonstrated that they can be combined with the immersion lens technology of VIGO system [23,24,25]. In this paper, we briefly describe the layout of the detector as a laterally-operated photoconductor, the superlattice and buffer layer growth as well as the detector processing. Then, after the detector is integrated into an IR detector module with a four-stage TE-cooler, which allows for operation at 200 K, we focus on a possible spectroscopy application in which an MCT-based IR detector module could be replaced by a T2SL-based IR detector module.

In addition to the cutoff wavelength, two more detector figures of merit are crucial for the content of this paper. The first is the specific detectivity $D^{*}$, which describes the signal-to-noise ratio:

(1) $$D^{*}\left(\lambda ,f\right)=\frac{R\left(\lambda \right)}{I_{n}\left(f\right)}\sqrt{A_{o}\Delta f}.$$

$D^{*}$ depends on the spectral responsivity R(λ), the noise current $I_{n}\left(f\right)$ and the bandwidth Δf. It is normalized to the optical detector area $A_{o}$. By using a lens to focus incoming radiation, $A_{o}$ can be increased significantly. The increase depends on the form of the lens and its refractive index n. A hyperhemispheric lens can increase $A_{o}$ by a factor of $n^{4}$ [26]. For backside-illuminated detectors, the lens can be immersed into the substrate material beneath the detector. The second figure of merit is the detector bandwidth that relates to the detection speed. For the device concept under study, the detector bandwidth is inversely proportional to the carrier recombination time. However, the responsivity is proportional to the carrier recombination time. Therefore, there is a trade-off between photosignal and detection speed in photoconductor optimization.

## 2. Design, Growth, Processing and Module Integration of an IR Detector

**Figure 2:**
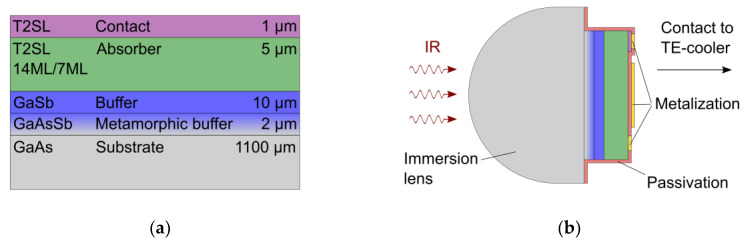
(**a**) Epitaxial layer structure for the fabrication of laterally-operated InAs/GaSb type-II superlattice (T2SL) detectors on GaAs substrate. (**b**) Schematic of a processed InAs/GaSb T2SL detector that is backside-illuminated through an immersion lens (not to scale).

The InAs/GaSb T2SL discussed in this paper was grown by molecular beam epitaxy on a 3 inch, n-type, (100)-oriented, 1100 µm thick GaAs substrate after careful calibration of shutter sequences and growth rates. Figure 2a shows the epitaxial layer structure. It consists of two buffer layers, the superlattice absorber layer and a thin superlattice contact layer. The first buffer layer is a metamorphic GaAsSb buffer, in which Sb gradually replaces As over 2 µm layer width. This results in a strain relaxed GaSb-like growth template for the subsequent layers [23]. The second buffer layer consists of 10 µm GaSb. This layer is followed by the superlattice absorber layer, which comprises 750 non-intentionally doped superlattice periods (residually n-type). Each of these periods features 14 monolayers (ML) InAs and 7 ML GaSb. InSb-like interfacial layers were realized between the individual InAs and GaSb sublayers to minimize the relative lattice mismatch to the underlying substrate. In the end, the heavily n-type doped contact layer was grown on top.

After growth, standard superlattice layer characterization was performed. A superlattice period length of 7.0 nm was determined by high-resolution X-ray diffraction, which was also used to verify the negligible relative lattice mismatch to the GaSb buffer. Spectral photoluminescence was measured to confirm the intended bandgap energy. At 10 K, a bandgap energy of 143 meV (corresponding to a wavelength of 8.7 µm) was obtained. As the bandgap shrinks for rising temperature, which can be described with the Varshni model [25], the corresponding cutoff wavelength of a detector increases. Hence, this superlattice can absorb radiation throughout a large fraction of the MIR at high operating temperatures.

Photolithographic processing was used to fabricate laterally-operated photoconductors (see Figure 2b). Unlike in most T2SL-based detector concepts, in which the current flows parallel to the superlattice growth direction, in this concept, the current flows mainly perpendicular to the growth direction between two ohmic metal contacts and requires external bias voltage for operation. When radiation of suitable wavelength enters the absorber layer, an additional photoconductivity is generated. The processing steps for detector fabrication included dry etching for structuring of the contact and the absorber layer, dielectric passivation, selective opening of the passivation layer and metalization. In the last step, the mesa front was also metalized. This metalized area acts as a mirror facilitating a double pass of the radiation incident from the backside, which increases the quantum efficiency. The processing sequence has been presented in more detail before [24].

A differing lattice constant of layer and substrate, which is the case for InAs/GaSb T2SLs lattice matched to GaSb on GaAs substrates, may result in an increased density of defects, growth inhomogeneities and a reduced device yield. Our wafer-level device characterization at 200 K suggested that device drop out due to material- or processing-related defects is negligible [24]. As device performance proved to be homogeneous across the wafer, a large device yield would be expected for manufacturing purposes. To allow for immersion of hyperhemispheric microlenses into the substrate, the detectors were processed with a horizontal and vertical pitch of 1480 µm. In this way, more than 1000 detectors could be fabricated per 3 inch wafer—the wafer size used in our study. Assuming the increasingly common 4 inch and 6 inch GaSb substrate diameters, the number of devices per wafer would scale according to the wafer area. For fabrication of detectors without substrate microlenses, the number of detectors per wafer depends on the intended detector size and can be significantly higher.

After processing and the characterization of the T2SLs and the fabricated detectors, the fully processed 3 inch wafers were diced into single element detectors. The module integration of the detector elements was completed in cooperation with VIGO System. In these modules, a T2SL detector 50 µm × 50 µm in size is mounted on top of a four-stage TE-cooler. The detectors feature a hyperhemispheric lens that was immersed into the GaAs substrate. As $n_{\mathrm{GaAs}}\approx \;$3.3, the lens increases $A_{o}$ for backside incident radiation by about two orders of magnitude and $D^{*}$ by one order of magnitude when compared to detector elements without such an immersion lens. Furthermore, the IR detector modules also comprise standard electronics from VIGO System: a fast preamplifier and a TE-cooler controller. These TE-cooled T2SL-based IR detector modules constitute RoHS-compatible turnkey systems.

## 3. Comparison to MCT

**Figure 3:**
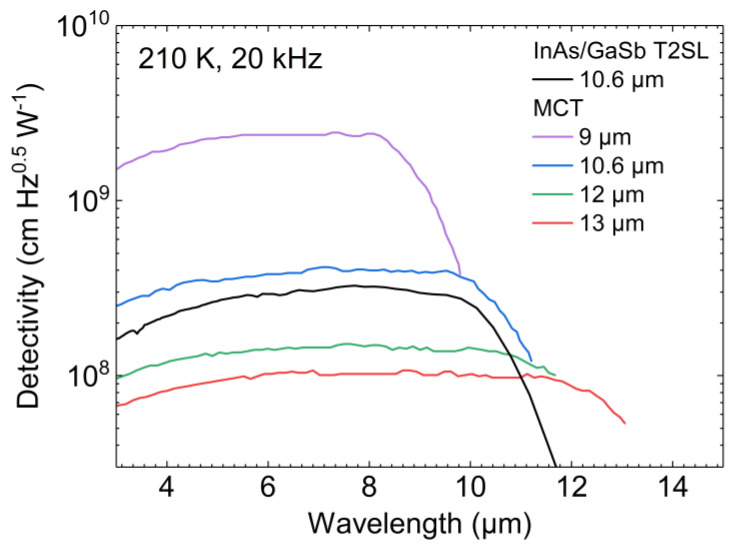
Detectivity of InAs/GaSb T2SL photoconductors (mean value) and commercial mercury cadmium telluride (MCT) photoconductors from VIGO System (guaranteed values) for different cutoff wavelengths at 210 K and 20 kHz [24,27].

To benchmark the performance of these detectors, we compare the detectivity of MCT-based and T2SL-based photoconductors without immersion lens. They are operated at 210 K with the noise current taken at 20 kHz. In Figure 3, we show the mean value of the detectivity of InAs/GaSb T2SL photoconductors, which we deduced from measurements that were already discussed in [24]. Here, we compare this mean value with specified detectivities of commercial MCT photoconductors from VIGO System for different cutoff wavelengths from 9–13 µm [27]. For detectors with the same cutoff wavelength of 10.6 µm, the detectivity of the MCT photoconductor is less than a factor of two higher than the detectivity of the T2SL photoconductors. Given the brief development of HOT InAs/GaSb T2SL photodetectors in comparison to the longstanding heritage of MCT photodetectors, this is a highly promising result. Doping optimization [25] and increasing the quantum efficiency are expected to further enhance the T2SL detector performance and increase its competitiveness.

As a longer cutoff wavelength implies a lower bandgap and an increased carrier generation, which leads to an increased noise level, the peak detectivity of InAs/GaSb T2SL detectors is expected to drop for longer cutoff wavelengths as it is the case for MCT-based detectors (see Figure 3). As the cutoff of an MCT detector crucially depends on the Cd content in the composition, which becomes more challenging to control precisely and homogeneously towards longer cutoff wavelength, for more elaborate device concepts the device yield drops and in turn the detector price rises. This drawback does not exist for InAs/GaSb T2SLs.

## 4. Real-Time MIR Backscattering Spectroscopy System

In addition to our development of HOT InAs/GaSb T2SL IR detectors, we realized a demonstrator system for MIR backscattering spectroscopy. The operation principle of the demonstrator exploits the characteristic spectral diffuse reflection of solid chemical substances in the MIR that can be utilized for substance identification. Using a fast spectrally tunable quantum cascade laser (QCL) as the illumination source and a fast photodetector, the system is able to record IR spectra over more than 250 cm^−1^ at rates of 1 kHz and therefore real-time spectroscopy. The high spectral scan speed of the system is ideal for fast changing scenarios or handheld operation as was demonstrated before. Here, we go beyond previous lab demonstrations of the measurement principle [28] as the system can run constantly without user intervention for several hours.

### 4.1. Setup of the Demonstrator System

**Figure 4:**
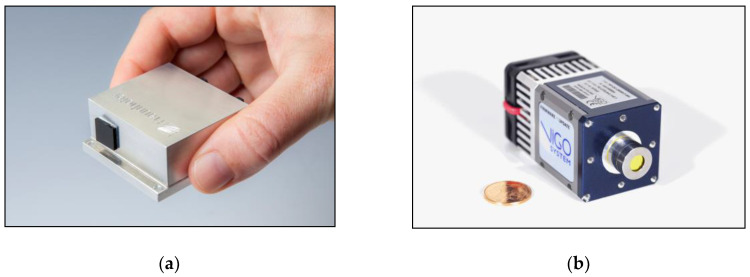
Photographs showing (**a**) the compact design*s* of the external cavity quantum cascade laser (EC-QCL) with micro-opto-electro-mechanical system (MOEMS) diffraction grating and (**b**) the high operating temperature (HOT) T2SL IR detector from Fraunhofer IAF in a detector module from VIGO System.

The first core component of the system, the IR light source, is an agile wavelength-tunable external cavity quantum cascade laser (EC-QCL) developed by Fraunhofer IAF and Fraunhofer IPMS [28,29,30]. Its emission wavelength is defined by the deflection of a resonant micro-opto-electro-mechanical system (MOEMS) diffraction grating in Littrow-configuration, which is driven close to the resonance frequency of ~1 kHz (i.e., it harmonically oscillates around its zero-deflection position). Synchronized with the MOEMS oscillation, the EC-QCL is operated in pulsed mode with a pulse length of 100 ns and a repetition rate of about 500 kHz. Due to the resonant nature of the MOEMS scanner, the laser wavelength is continuously tuned and the full spectral range between 1060 cm^–1^ and 1350 cm^−1^ provided by the QCL chip can be scanned in only half a MOEMS period, i.e., ~500 µs. However, typically the IR spectra are constructed from a full MOEMS period, as this increases the spectral resolution. For the parameters mentioned above, one achieves a typical spectral resolution of about 2 cm^−1^ and a spectral broadening per pulse (i.e., per emission wavelength) also of <2 cm^−1^ [27]. These performance parameters allow for spectroscopy on a number of solids and liquids with characteristic bands within the IR fingerprint region. The laser module itself is very compact, as can be seen in Figure 4a. Fraunhofer IAF and IPMS have also developed a non-resonant MOEMS EC-QCL, which allows addressing individual wavelength or (arbitrary) trajectories with scan frequencies of up to several ten hertz in an identical footprint [31].

The second core component of the system, a fast IR photodetector, detects the QCL radiation after it is diffusely reflected by the substance under investigation. The detector was chosen to meet the requirements set by the laser system. These were a MHz-bandwidth, to resolve each individual laser pulse, and a sufficiently long cutoff wavelength, to cover the required spectral range. As in diffuse reflection typical signal intensities are small, a high D* is also necessary. To achieve a portable and compact system, only TE-cooled detectors were considered. Up until now, only MCT detectors met these requirements and hence an MCT-based IR detector module was initially selected. Its specifications will be presented later, alongside those of the T2SL-based IR detector module. It differs from an MCT-based IR detector module from VIGO only in terms of the employed detector chip. Figure 4b demonstrates the small size of the detector module.

**Figure 5:**
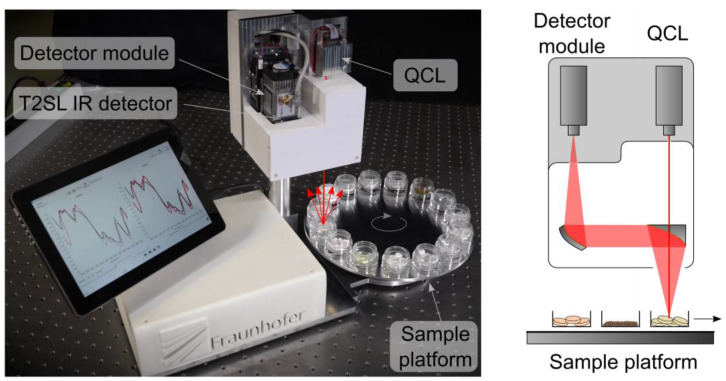
Picture and simplified schematic of the demonstrator system. Backscattering IR spectra are continuously recorded using the tunable EC-QCL and the HOT InAs/GaSb T2SL IR detector. As the sample platform rotates, different substances are illuminated and subsequently identified after comparison with the database.

A picture of the demonstrator system and a simplified schematic showing its interior are presented in Figure 5. During the operation of the system, the QCL beam impinges on a continuously rotating sample platform. On this platform, several substances in the form of pills, powders or foils are arranged in small sample compartments. The samples are listed in Table 1.

As the sample platform rotates, the different substances are sequentially exposed to the incoming QCL beam. The rotational frequency of the sample platform sets the exposure time per substance. In our case, the sample platform rotates with a speed of ~4 rpm, resulting in an exposure time per substance of around ~1 s. After interaction with the respective substance, the laser radiation diffusely backscatters. In the case of the foils, the transmitted light is diffusely backscattered by a plate located below them. Then, the collected portion of the backscattered light is deflected and focused to the fast IR detector. Each single laser pulse is detected, and an IR spectrum is constructed. The substance identification occurs by matching the measured fingerprint spectra to the previously acquired database spectra. The realization of the identification process is described in more detail in the following section.

### 4.2. System Operation and Database Comparison

Each spectrum measured with the demonstrator system contains a spectral signature, mainly due to the wavelength dependence of the responsivity of the detector. To determine the reflectivity of the different substances under test, the system-dependent spectral signature needs to be corrected for. Hence, at the beginning of the experiment a reference spectrum is acquired with a diffuse scattering plate. It is placed at the same distance as the rotating samples on the sample platform. During operation of the demonstrator system, the measured spectra are always divided by this reference.

With the demonstrator system, IR spectra are continuously recorded at a rate of 1 kHz. Typically, 25 spectra are averaged, corresponding to only 25 ms measurement time. Subsequently, the averaged spectra are compared to a database by using a cross-correlation algorithm that enables substance identification. The database is composed of MIR diffuse reflection spectra (in the case of pills or powders) or transmission spectra (in the case of foils). These spectra were previously acquired with the system itself or a commercial FTIR spectrometer from the same samples. Note that a FTIR measurement takes several minutes in order to achieve a spectral point spacing (~2 cm^−1^) comparable to our MOEMS EC-QCL-based measurement.

The averaged spectra are continuously compared to the database, while new spectra are still acquired within that time. Hence, no time is lost due to the post-processing of data. Regarding the comparison algorithm, we chose a standard cross-correlation comparison algorithm for simplicity, which is explained in more detail below. The idle time of the system between the recordings of two averaged spectra is sufficient to perform a comparison with the database. The database comprises 15 substances in the given case, which enables a comparison in ~10 ms when using this algorithm. The same algorithm could also be employed for an enlarged database; however, it would be at the cost of a slower database comparison.

In detail, we use the following procedure in our analysis. First, we calculate the normalized cross correlation (NCC) of the averaged spectrum to each database entry. It serves as measure for the similarity between two sets of data. Then, the largest cross-correlation (NCC_max_) is selected. If NCC_max_ is larger than a threshold value (NCC_th_), the substance related to the respective database entry is considered as identified. Thereupon, the name of the substance is displayed on the demonstrator screen together with the averaged and the database spectrum. If NCC_max_ is smaller than NCC_th_, no output is returned. It needs to be mentioned that NCC_th_ is an arbitrary yet fixed number. It is chosen based on the measurement conditions at the beginning of the experiment after an initial test run. It is set as high as possible in order to avoid false positives and as low as possible in order to avoid no returns.

The post-processing of data does not need to interfere with the acquisition of further spectra. To save time, this part can be delegated to different sub-systems or processors on demand, i.e., to a distributed computing architecture. This would also allow a more advanced data processing and analysis. In this context, resolving mixtures into their components or an automatized subtraction of spectral fingerprints from their background are typically of interest. Background subtraction becomes particularly important for the analysis of samples that are not bulk-like, e.g., when a potentially hazardous powder sample on an unknown substrate needs to be identified [32,33].

The presented approach for substance identification or discrimination with the demonstrator setup is solely based on matching IR fingerprint spectra to database spectra that were previously measured for known substances. Therefore, no precise knowledge about the specific nature of the vibrational or vibrational-rotational molecular bands is needed for identification. In fact, the set of substances on the sample platform was chosen arbitrarily. A modified set of substances could also be used as long as the corresponding spectra provide sufficient distinction for discrimination in the MIR.

### 4.3. Detector Module Interchangeability

To demonstrate the applicability of the T2SL-based IR module for spectroscopy, we replaced the MCT-based IR detector module in the demonstrator system with the T2SL-based IR detector module. The MCT-based module features a two-stage TE-cooled, photovoltaic IR detector that is illuminated via a hemispheric lens resulting in an optical area of 1 mm × 1 mm. This module has been specified with a cutoff wavelength of 10.6 µm, a bandwidth of 100 MHz and a detectivity of 6.8 × 10^8^ cm$\sqrt{\mathrm{Hz}}$/W. The T2SL-based module features a four-stage TE-cooled, photoconductive, 50 µm × 50 µm-sized IR detector that is illuminated via a hyperhemispheric lens, resulting in an optical area of approximately 500 µm × 500 µm. It has been specified with a cutoff wavelength of 9.3 µm, a bandwidth of 10 MHz and a detectivity of 6.7 × 10^9^ cm$\sqrt{\mathrm{Hz}}$/W. The specifications of both detectors are listed in Table 2.

As both modules feature equal packages and housings from VIGO System, replacing the MCT-based detector module, integrating the T2SL-based IR detector module into the setup and its optical alignment were straightforward. In the following, we report on the operation of the demonstrator system with both IR detector modules.

**Figure 6:**
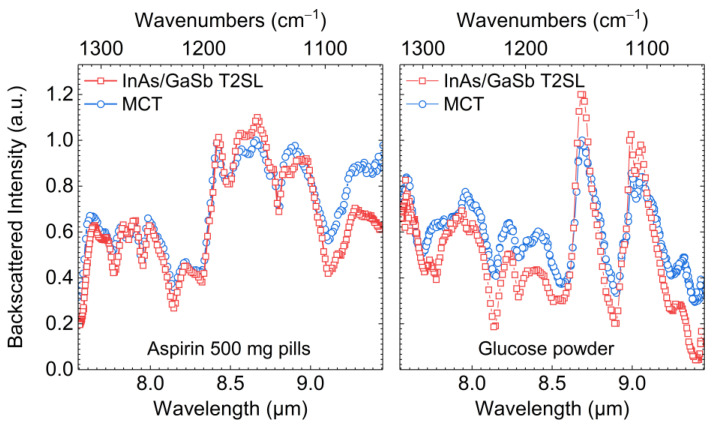
Diffuse IR reflectance spectra obtained from aspirin 500 mg pills and glucose powder with the two IR detector modules featuring a HOT InAs/GaSb T2SL IR detector and a HOT MCT IR detector during operation of the demonstrator system.

In Figure 6, exemplary diffuse reflection spectra are shown, which were measured during operation of the demonstrator system on commercial aspirin 500 mg pills and glucose powder with the T2SL-based and MCT-based IR detector module, respectively. The spectra measured with the MCT detector are normalized to their maximum value. The spectra acquired with the T2SL detector are multiplied by a constant, chosen to simplify comparison of the spectra. For both substances, the spectral trends measured with the two IR detectors are well comparable. Clearly, both detectors were able to resolve characteristic spectral features of the aspirin pills and the glucose powder, which allowed for substance identification by database comparison. As all substances on the sample platform (Table 1) have characteristic spectral features in the spectral range coverable with both IR detector modules, the T2SL-based IR detector module was also able to identify them during standard operation after fast comparison with the database in real-time.

### 4.4. Long-Term Stabilty

We studied the long-term stability of the demonstrator system with the T2SL detector. Following the identification procedure described before, we recorded the identified substance and the calculated NCC as a function of time. More than 50,000 measurements were performed in over 16 h of measurement time. Since the rotation speed of the sample platform is not constant, but rather fluctuates constantly in an uncontrolled manner, on average a new substance was identified every 1.1 ± 0.25 s. As there is no synchronization between the platform and our laser system, for each averaged spectrum, a different area was illuminated and used for analysis.

**Figure 7:**
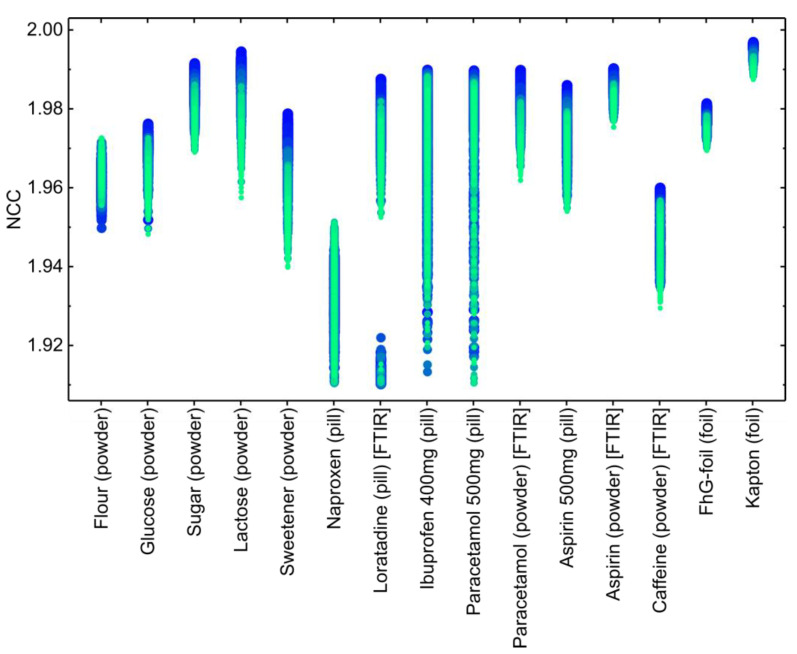
Investigation of the long-term stability of the demonstrator system using the T2SL detector. The time information is encoded in color and size of the dots, i.e., early results are represented by big blue dots, whereas later results are given by smaller and greener dots. Please note the two groups for loratadine, where the lower ones correspond to false assignments.

The results of the long-term stability test of the substance identification are presented in Figure 7. The time evolution is encoded in the figure through the color and size of the dots that are used to represent a single result. Substances for which the reference spectrum was obtained by the FTIR spectrometer are labelled accordingly. Overall, no significant drifts can be observed in the data. However, the distribution of the NCC strongly depends on the substance and varies depending on its form, i.e., foil, pill, or powder. In general, the transmission spectra through the foils show narrower distributions, whereas for the pills the distributions are typically wider. The assumption of our simple model—that each substance can be matched to the database using a single database spectrum—does not necessarily hold for the pills. This relates to the difficulties in solid dose manufacturing to achieve good homogeneity in the blending process. This also broadens the distributions of NCC values in our analysis.

The detailed analysis of the results of the long-term stability test with more than 50,000 measurements showed that in total 16 samples could not be identified and 68 measurements have been assigned to the wrong substance. Furthermore, 67 of these events correspond to a false assignment to loratadine. Since the corresponding NCCs of these events are smaller and form a separate group in Figure 7, these events could easily be rejected, if a more complex model were used. The 16 missing hits are attributed to ibuprofen 400 mg and the naproxen-based pill, certainly due to a too high NCC_th_ value, which was chosen to be 1.91 in this experiment. In total, the error rate for missing hits is as low as 0.3‰ with the potential to be improved.

## 5. Discussion

The detector development and the presented application show the potential of TE-cooled T2SL-based IR detector modules for substance discrimination and in a broader scope for IR spectroscopy in general. This potential results from several key properties that the IR detector module exhibits. The first key property is a sufficiently long cutoff wavelength, which is tunable for InAs/GaSb T2SL detectors as it is for MCT detectors. The other key properties are a high detectivity and a bandwidth in the MHz range. A meaningful one-to-one comparison of the two IR detector modules used in the demonstrator system is problematic as they differ in several specifications such as size, operation mode, operating temperature, and cutoff wavelength (see Figure 3). The properties of the T2SL-based IR detector module can be slightly altered by changing the cooling power and hence the operating temperature. Rising the operating temperature of the InAs/GaSb T2SL photoconductor increases the cutoff wavelength and the detector bandwidth but reduces the detectivity. Due to the versatility of the InAs/GaSb T2SL material system and mature device processing at hand, detectors operating in more elaborate device concepts and with properties tailored to a particular application could be realized for the HOT range.

## 6. Summary

We demonstrated a RoHS-compatible, TE-cooled IR detector module based on an InAs/GaSb T2SL single element detector. For the fabrication of this module, we combined Fraunhofer IAF’s expertise in the growth and processing of InAs/GaSb T2SLs with VIGO System’s expertise in the fabrication of TE-cooled IR detector modules. This paper shows that this T2SL-based IR detector module and a commercial MCT-based IR detector module can be employed interchangeably in a compact and real-time MIR backscattering spectroscopy system. This system provides a very low error rate of only 0.3‰ in substance differentiation, which can be further improved. Furthermore, we showed that for equal operation mode, operating temperature, cutoff wavelength, and noise frequency, the detectivity of photoconductors based on InAs/GaSb T2SLs and MCT is comparable. This renders InAs/GaSb T2SLs promising for fully RoHS-compatible HOT IR photodetectors.

## Figures and Tables

**Table 1:** Samples used in the demonstrator system.

Presentation	Sample
pills	Naproxen, Loratadine, Ibuprofen 400 mg, Paracetamol 500 mg, Aspirin 500 mg
powder	Flour, Glucose, Sugar, Lactose, Sweetener, Paracetamol, Aspirin, Caffeine
foil	Kapton, FhG-foil (sticky tape)

**Table 2:** Specifications of the two IR detector modules, based on an MCT detector and an InAs/GaSb T2SL detector, respectively.

	MCT	InAs/GaSb T2SL
Detectivity	6.8 × 10^8^ cm$\sqrt{\mathrm{Hz}}$/W	6.7 × 10^9^ cm$\sqrt{\mathrm{Hz}}$/W
Bandwidth	100 MHz	10 MHz
Cutoff Wavelength	10.6 µm	9.3 µm
Operating temperature	226 K (2-stage TEC)	200 K (4-stage TEC)
Operation mode	photovoltaic	photoconductive
Optical Area	1 mm × 1 mm	0.5 mm × 0.5 mm
Lens	hemispheric	hyperhemispheric

## References

[1] Lambrecht A., Schmitt K., Tournié E., Cerutti L. (2007). Mid-infrared gas-sensing systems and applications. Mid-Infrared Optoelectronics: Materials, Devices, and Applications.

[2] Schwaighofer A., Brandstetter M., Lendl B. (2017). Quantum cascade lasers (QCLs) in biomedical spectroscopy. Chem. Soc. Rev..

[3] Piotrowski J., Galus W., Grudzien M. (1991). Near room-temperature IR photo-detectors. Infrared Phys..

[4] Kalinowski P., Mikołajczyk J., Piotrowski A., Piotrowski J. (2019). Recent advances in manufacturing of miniaturized uncooled IR detection modules. Semicond. Sci. Technol..

[5] Directive 2011/65/EU of the European Parliament and of the Council “RoHS-Restriction”. https://eur-lex.europa.eu/legal-content/En/TXT/?uri=celex%3A32011L0065.

[6] Murawski K., Gomolka E., Kopytko M., Grodecki K., Michalczewski K., Kubiszyn L., Gawron W., Martyniuk P., Rogalski A., Piotrowski J. (2019). Bandgap energy determination of InAsSb epilayers grown by molecular beam epitaxy on GaAs substrates. Prog. Nat. Sci. Mater. Int..

[7] Sai-Halasz G.A., Tsu R., Esaki L. (1977). A new semiconductor superlattice. Appl. Phys. Lett..

[8] Sai-Halasz G.A., Esaki L., Harrison W.A. (1978). InAs-GaSb superlattice energy structure and its semiconductor-semimetal transition. Phys. Rev. B.

[9] Sai-Halasz G.A., Chang L., Welter J.-M., Chang C.-A., Esaki L. (1978). Optical absorption of In1 xGaxAs/GaSb1 yAsy superlattices. Solid State Commun..

[10] Masur J.-M., Rehm R., Schmitz J., Kirste L., Walther M. (2013). Four-component superlattice empirical pseudopotential method for InAs/GaSb superlattices. Infrared Phys. Technol..

[11] Smith D.L., Mailhiot C. (1987). Proposal for strained type II superlattice infrared detectors. J. Appl. Phys..

[12] Herres N., Fuchs F., Schmitz J., Pavlov K.M., Wagner J., Ralston J.D., Koidl P., Gadaleta C., Scamarcio G. (1996). Effect of interfacial bonding on the structural and vibrational properties of InAs/GaSb superlattices. Phys. Rev. B.

[13] Umana-Membreno G.A., Klein B., Kala H., Antoszewski J., Gautam N., Kutty M.N., Plis E., Krishna S., Faraone L. (2012). Vertical minority carrier electron transport in p-type InAs/GaSb type-II superlattices. Appl. Phys. Lett..

[14] Johnson J.L., Samoska L.A., Gossard A.C., Merz J.L., Jack M.D., Chapman G.R., Baumgartz B.A., Kosai K., Johnson S.M. (1996). Electrical and optical properties of infrared photodiodes using the InAs/Ga1-xInxSb superlattice in heterojunctions with GaSb. J. Appl. Phys..

[15] Fuchs F., Weimer U., Pletschen W., Schmitz J., Ahlswede E., Walter M., Wagner J., Koidl P. (1997). High performance InAs/Ga1-xInxSb superlattice infrared photodiodes. Appl. Phys. Lett..

[16] Ting D., Hill C.J., Soibel A., Keo S.A., Mumolo J.M., Nguyen J., Gunapala S.D. (2009). A high-performance long wavelength superlattice complementary barrier infrared detector. Appl. Phys. Lett..

[17] Walther M., Rehm R., Fuchs F., Schmitz J., Fleißner J., Cabanski W., Eich D., Finck M., Rode W., Wendler J. (2005). 256 × 256 focal plane array midwavelength infrared camera based on InAs/GaSb short-period superlattices. J. Electron. Mater..

[18] Ting D., Soibel A., Höglund L., Nguyen J., Hill C.J., Khoshakhlagh A., Gunapala S.D., Gunapala S.D., Rhiger D.R., Jagadish C. (2011). Type-II Superlattice Infrared Detectors. Semiconductors and Semimetals.

[19] Mohseni H., Litvinov V.I., Razeghi M. (1998). Interface-induced suppression of the Auger recombination in type-II InAs/GaSb superlattices. Phys. Rev. B.

[20] Mohseni H., Razeghi M. (2001). Long-wavelength type-II photodiodes operating at room temperature. IEEE Photonics Technol. Lett..

[21] Mohseni H., Michel E., Sandoen J., Razeghi M., Mitchel W., Brown G. (1997). Growth and characterization of InAs/GaSb photoconductors for long wavelength infrared range. Appl. Phys. Lett..

[22] Mohseni H., Wojkowski J., Razeghi M., Brown G., Mitchel W. (1999). Uncooled InAs-GaSb type-II infrared detectors grown on GaAs substrates for the 8-12-μm atmospheric window. IEEE J. Quantum Electron..

[23] Müller R., Gramich V., Wauro M., Niemasz J., Kirste L., Daumer V., Janaszek A., Jureńczyk J., Rehm R. (2019). High operating temperature InAs/GaSb type-II superlattice detectors on GaAs substrate for the long wavelength infrared. Infrared Phys. Technol..

[24] Müller R., Niemasz J., Daumer V., Janaszek A., Jureńczyk J., Rehm R. (2019). Advances on photoconductive InAs/GaSb type-II superlattice long-wavelength infrared detectors for high operating temperature. Proc. SPIE.

[25] Müller R., Niemasz J., Daumer V., Rehm R. Design guidelines for high operating temperature InAs/GaSb type-II superlattice photoconductors for the longwave infrared.

[26] Piotrowski J., Rogalski A. (2007). High-Operating-Temperature Infrared Photodetectors.

[27] VIGO System Homepage. https://vigo.com.pl/produkty/pc-3te/.

[28] Butschek L., Hugger S., Jarvis J., Härtelt M., Merten A., Schwarzenberg M., Grahmann J., Stothard D.M., Warden M., Carson C. (2017). Microoptoelectromechanical systems-based external cavity quantum cascade lasers for real-time spectroscopy. Opt. Eng..

[29] Grahmann J., Merten A., Ostendorf R., Fontenot M., Bleh D., Schenk H., Wagner H.-J. (2014). Tunable External Cavity Quantum Cascade Lasers (EC-QCL): An application field for MOEMS based scanning gratings. Proc. SPIE.

[30] Ostendorf R., Butschek L., Hugger S., Fuchs F., Yang Q., Jarvis J., Schilling C., Rattunde M., Merten A., Grahmann J. (2016). Recent advances and applications of external cavity-QCLs towards hyperspectral imaging for standoff detection and real-time spectroscopic sensing of chemicals. Photonics.

[31] Haertelt M., Hugger S., Butschek L., Schilling C., Merten A., Schwarzenberg M., Dreyhaupt A., Grahmann J., Rattunde M., Ostendorf R. (2019). Advances of MOEMS-based external cavity QCLs. Proc. SPIE.

[32] Jarvis J., Fuchs F., Hugger S., Ostendorf R., Butschek L., Yang Q., Dreyhaupt A., Grahmann J., Wagner J. (2016). Hyperspectral image analysis for standoff trace detection using IR laser spectroscopy. Proc. SPIE.

[33] Jarvis J., Haertelt M., Hugger S., Butschek L., Fuchs F., Ostendorf R., Wagner J., Beyerer J. (2017). Hyperspectral data acquisition and analysis in imaging and real-time active MIR backscattering spectroscopy. Adv. Opt. Technol..

